# Advection and diffusion in perivascular and extracellular spaces in the brain

**DOI:** 10.1098/rsif.2025.0010

**Published:** 2025-05-21

**Authors:** Yisen Guo, Keelin Quirk, Douglas H. Kelley, John H. Thomas

**Affiliations:** ^1^ Mechanical Engineering, University of Rochester, Rochester, NY, USA

**Keywords:** brain solute clearance, cerebrospinal fluid flow, glymphatic system, perivascular spaces, advection, diffusion

## Abstract

Knowledge of the relative importance of advection and diffusion in clearing waste from the brain has been elusive, especially concerning the extracellular space (ECS). With local and global computational models of the mouse brain, we explore how the presence or absence of advection in the ECS affects solute transport. Without advection in the ECS, clearance would occur by diffusion into flowing cerebrospinal fluid in perivascular spaces (PVSs) or elsewhere, but we find this process to be severely limited by build-up of solute in the PVSs. We simulate flow in the ECS driven by a pressure drop between arteriole and venule PVSs, which enhances clearance considerably. To assess the relative importance of advection and diffusion, we introduce a *local* Péclet number P(x,t), a dimensionless scalar field. For our simulations, P≪1 through much of the ECS but P≥1 near PVSs near the brain surface. This local dominance of advection in the ECS establishes a clearance mechanism markedly different from that produced by diffusion alone. In network simulations that explore different parameter values and efflux routes, the pressures needed to drive the PVS flows measured *in vivo* are unrealistically large for most cases lacking ECS flow. Collectively, our models indicate that a flow in the ECS is necessary to explain experimental measurements and maintain homeostasis.

## 1. Introduction

There are no lymph vessels in the interior of the brain: the removal of metabolic waste molecules is instead accomplished by advection and diffusion within the interstitial fluid (ISF) filling the extracellular space (ECS) and cerebrospinal fluid CSF filling the perivascular spaces (PVSs) that surround the blood vessels (arterioles, venules and perhaps capillaries) [1,2]. The details of this waste-clearance system are not well understood, and in particular, the relative importance of advection and diffusion in the system is still controversial (see recent reviews [3–5]). Diffusion of solutes in the porous ECS is well understood, thanks to the extensive work by Nicholson and others [6–10], which showed that the effective diffusion coefficient is set by ECS tortuosity. Here, and throughout, we consider the ECS excluding perivascular and intravascular spaces. Assessing the contributions of advection requires detailed knowledge of the velocity fields of CSF and ISF: while such details are being revealed for CSF flow in surface PVSs [11–14], they are lacking for flows in the interior of the brain, particularly in the ECS.

Here we consider the possible ways in which advection and diffusion could contribute to brain clearance under different scenarios. In one scenario, there is no flow of CSF or ISF anywhere within the brain, so there is no advection and clearance is by diffusion alone. We mention this scenario, in spite of the fact that there is strong evidence for flow of CSF along penetrating PVSs and considerable evidence that there may be flow of ISF in the ECS, because doubts have been expressed about the existence or importance of any such flow (e.g. [15–17]). Diffusion is a slow process, and it is hard to imagine that evolution would leave one of the largest organs in the body, with one of the highest metabolic rates, with such an inefficient means of clearing metabolic waste. This inefficiency is illustrated by a simple spherical brain model [5], which shows how diffusion acting alone will produce a highly inhomogeneous distribution of a metabolic waste solute, with very high concentrations at the centre of the brain. We dismiss this scenario and shall not consider it further in this paper. Instead, we consider the following two scenarios.

*Scenario A*: There is a flow of CSF along the network of PVSs in the interior of the brain but no flow of ISF in the rest of the parenchyma. Clearance is by diffusion of a solute from the ECS into PVSs, where the solute is then advected out of the brain along a network of PVSs. This scenario was discussed briefly by one of us [18], pointing out how it might work provided that there are continuous pathways for CSF flow along PVSs surrounding penetrating arteries, arterioles, capillaries, venules and veins. The existence of such continuous PVS pathways has been neither firmly established nor firmly refuted. Here we examine this scenario with different models. In particular, we seek to assess the effect of the build-up of solute in the CSF flowing through the network of PVSs, which reduces the rate at which a solute diffuses from the ECS into the PVSs, an effect that has received little attention in previous studies.

*Scenario B*: There is a flow of CSF along the network of PVSs in the interior of the brain and also a slow flow of ISF in the ECS in the rest of the parenchyma, and solutes are carried by both advection and diffusion to PVSs along venules and then carried to the lymphatic system. This scenario is supported by the early experimental findings of Cserr *et al*. [19] and also corresponds to the ‘glymphatic system’ as originally proposed [20]. The hypothesized slow flow of ISF is very difficult to measure directly, and its existence has proved to be controversial. Simulations by Holter *et al*. [21] indicated that the permeability of the ECS is too low to allow for any substantial flow of ISF. However, several other, more recent pieces of evidence indicate the existence of a slow flow of ISF in the ECS and hence at least some advective transport there. This evidence includes results from modelling of experimental data [22–26], which show a better fit to tracer data when a slow flow of ISF is included. There is a theoretical argument for a flow of ISF [27] based on the observed increase in tissue porosity and solute clearance from the awake state to the sleep state [28]. Also, a recently proposed mechanism for producing a directed flow in PVSs driven by arterial pulsations, in which the glial endfoot gaps in the wall of a PVS act as valves, necessarily drives a flow of ISF in the ECS [29–31].

## 2. Mathematical aspects of advection and diffusion in the brain

The basic equation governing the concentration C(x,t) of a passive solute in the brain is the *advection–diffusion equation* [5,32]


(2.1) $$\frac{\partial C}{\partial t}+\text{u}\cdot \nabla C=D\nabla^{2}C+f,$$

where u is the Eulerian velocity field (referred to a fixed frame of reference), D is the diffusion coefficient, f is the source term (the rate of generation of the solute per unit volume per unit time), t is time, ∇ is the spatial gradient operator and $\nabla^{2}$ is the Laplacian operator. This form of the advection–diffusion equation assumes a homogeneous, incompressible fluid and uniform, isotropic diffusivity D. Of course the brain parenchyma is not homogeneous: it consists of a porous network of neurons and supporting tissue filled with ISF, and we are interested in solute transport within the ISF. On length scales larger than the microstructure, we can treat the medium as homogeneous if we replace D with an effective diffusion coefficient that accounts for the volume fraction occupied by the fluid and the jagged paths that diffusing molecules must follow, represented by the *tortuosity*. There is an extensive literature devoted to justifying this approach and determining effective diffusivities experimentally: see, for example [8,9,33]. Here we shall assume throughout that D represents an effective diffusion coefficient.

In applying the advection–diffusion equation to models of the transport of solutes in the brain, the velocity field will either be specified or calculated separately, thus decoupling the advection–diffusion equation from the fluid-dynamic equations. Coupling of the equations would occur if we considered the effects of osmosis, which we do not, or if the viscosity of the fluid changed substantially with changes in the concentration of the solute, which we assume is not the case (this is a good assumption for CSF and ISF [34]).

The combined effects of advection and diffusion produce *dispersion* of a solute. The relative importance of advection and diffusion is usually measured by a dimensionless number, the *Péclet number* Pe, which estimates the ratio of the magnitudes of the advection and diffusion terms in equation (2.1):


(2.2) $$\frac{|\text{u}\cdot \nabla C|}{\left|D\nabla^{2}C\right|}∼\frac{UC_{0}/L}{D\left(C_{0}/L^{2}\right)}∼\frac{UL}{D}\equiv Pe,$$

where U is a velocity scale, $C_{0}$ is a typical value of the concentration and L is a length scale for spatial variations in the concentration. For the flows observed in the PVSs of arterioles in the mouse brain [11,12], the Péclet number is large: Pe∼1000 for the microspheres used in the experiments and Pe∼10−100 for other solutes of interest. In these perivascular flows advection dominates diffusion. However, we do not expect this to be the case throughout the entire system: the flow branches into very many smaller PVSs along arterioles, capillaries, venules and veins, and hence is much slower in these channels. If there is bulk flow of ISF through the ECS, it is likely to be slower still, and Péclet numbers of less than unity are to be expected.

The Péclet number defined in equation (2.2) is a global quantity, having a single value for the region in question, based on suitable fixed values of the velocity scale U and length scale L. However, the relative values of the advection and diffusion terms in equation (2.1) can vary substantially in space and time in a given region, and they also can act in opposition. For equation (2.1) written in the form


(2.3) $$\frac{\partial C}{\partial t}=-\text{u}\cdot \nabla C+D\nabla^{2}C+f,$$

it is useful to define a *local Péclet number* P(x,t) as the ratio of the advection and diffusion terms on the right-hand side:


(2.4) $$P(\text{x},t)\equiv \frac{-\text{u}\cdot \nabla C}{D\nabla^{2}C}.$$

This local Péclet number is a scalar field that can vary in space and time, and it is also a signed quantity that is positive when advection and diffusion are both acting in the same sense, to either reduce or increase the local solute concentration. Note that advection tends to decrease the local concentration when u has a component in the direction of the solute gradient (i.e. when u⋅∇C>0) and tends to increase the local concentration when u has a component in the direction opposite that of the concentration gradient (i.e. when u⋅∇C<0). The fact that there can be regions where advection and diffusion act in opposition makes this signed Péclet number a much better indicator of the local situation than a strictly positive one defined as a ratio of magnitudes. Also, as we shall see in the simulations presented here, this local Péclet number can vary by several orders of magnitude over the domain, it can be either positive or negative, and advection can substantially affect the character of solute dispersion even when the magnitude of the local Péclet number is larger than unity only in small subregions of the domain.

Solute transport is effected by the sum $\text{J}={\text{J}}_{\text{A}}+{\text{J}}_{\text{D}}$ of the advective flux ${\text{J}}_{\text{A}}=\text{u}C$ and the diffusive flux ${\text{J}}_{\text{D}}=-D\nabla C$. We can arrive at the advection–diffusion equation (2.1) by setting the local time rate of change of the concentration C equal to the negative divergence of the total flux plus the local rate of production f:


(2.5) $$\frac{\partial C}{\partial t}=-\nabla \cdot \text{J}+f=-\nabla \cdot (\text{u}C)-\nabla \cdot (-D\nabla C)+f=-\text{u}\cdot \nabla C+D\nabla^{2}C+f,$$

noting that mass conservation requires that ∇⋅u=0 for the incompressible fluid and that the diffusivity D is assumed to be uniform (∇D=0). Although the local rate of change of concentration is determined by the divergence of the fluxes ${\text{J}}_{\text{A}}$ and ${\text{J}}_{\text{D}}$, not by the fluxes themselves, it is of some interest to consider the magnitude and direction of each of these fluxes at various points in the domain. The directions are conveniently represented by unit vectors ${\text{e}}_{\text{A}}\equiv \text{u}/|\text{u}|$ and ${\text{e}}_{\text{D}}\equiv -\nabla C/|\nabla C|$. The fluxes ${\text{J}}_{\text{A}}$ and ${\text{J}}_{\text{D}}$ are in general in different directions, and their alignment is conveniently represented be the scalar product of their unit vectors, ${\text{e}}_{\text{A}}\cdot {\text{e}}_{\text{D}}$. The values of this scalar quantity lie in the range $-1\leq {\text{e}}_{\text{A}}\cdot {\text{e}}_{\text{D}}\leq 1$, and the limiting values −1 and 1 correspond to fluxes aligned in the opposite or same direction, respectively.

In modelling advection and diffusion within the CSF and ISF in the brain, there are two basic and quite different types of mathematical problems of interest. In simple terms, these problems can be described as follows:

*Problem 1*. Here the aim is to model the observed time-varying concentration distribution C(x,t) in an experiment in which a tracer solute or drug is injected into the brain. This is an initial-boundary-value problem, in which there is no internal production of the solute, but instead an initial concentration of the solute is specified. In this case, for example, one solves the time-dependent advection–diffusion equation (2.1) for a specified steady velocity field u(x), a specified initial concentration $C(\text{x},0)=C_{0}(\text{x})$, no source term (f=0), and suitable model geometry and boundary conditions. Alternatively, an injection taking place over an extended time can be represented by a source term f(x,t). The problem can also be posed for a known time-dependent velocity field u(x,t).

*Problem 2*. This is a steady-state problem, to determine the distribution of a naturally occurring metabolic waste solute, produced at a steady rate, and advected by a known velocity field of the fluid. In this case, one solves equation (2.1) with ∂C/∂t=0, a specified steady velocity field u(x), and a given steady source term f(x). This problem is aimed at understanding the brain’s actual clearance mechanism for metabolic waste, which must maintain a steady-state concentration (on average, subject to fluctuations associated with sleep/wake changes and other variations in brain state) in order to prevent the build-up of metabolic waste and maintain homeostasis.

Problem 2 is of course the fundamental one, describing the actual working of the brain’s mechanism for clearing metabolic waste, but Problem 1 has been much studied because it relates directly to experimental observations of the transport of an injected tracer. We shall consider both types of problems in the models we present here, and point out some important differences.

## 3. Local models of clearance by advection and diffusion

In this section, we present local models of transport based on scenarios A and B. The simplest model consists of a single penetrating arteriole, with a flow of CSF in its PVS, and a surrounding region of the parenchymal ECS from which a solute is cleared. We follow this with a more complete model consisting of an array of arteriole and venule PVSs within an extended region of the parenchyma. In Scenario A, these models show that high solute concentration along PVSs slows diffusive clearance from the surrounding parenchyma. In Scenario B, these models show how clearance can be enhanced by a flow of ISF.

The numerical methods used in computing the velocity and concentration fields in these local models are described in appendix A.

### 3.1. A single perivascular space and its surrounding extracellular space

**Figure 1.**
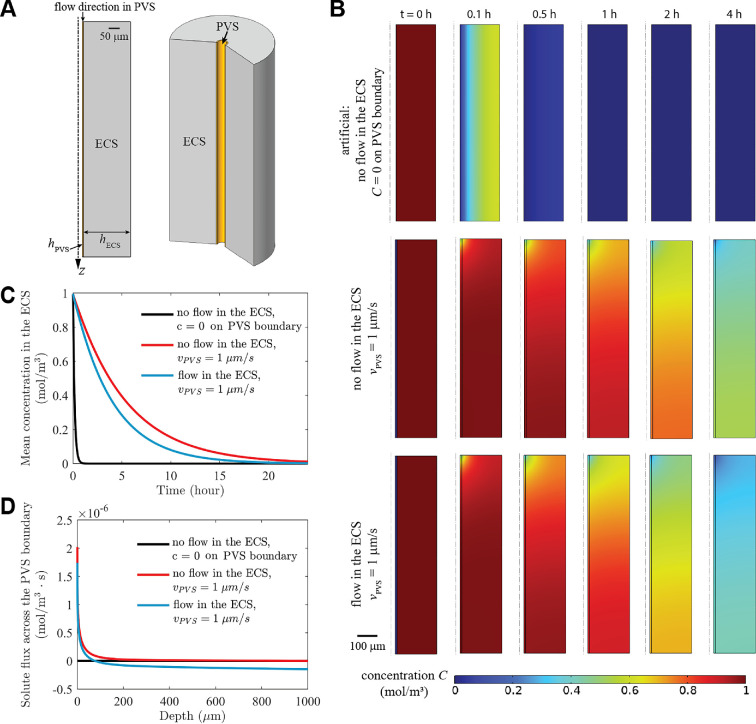
Time-dependent (Problem 1) numerical simulations for a single PVS and its surrounding ECS. (A) Computational domain showing the widths of the PVS and ECS and the flow direction in the PVS. The solute concentration in the ECS is initially uniform, C=1 at t = 0. (B) Concentration distributions at various times for three simulations. Top row: no flow in the ECS, concentration maintained at zero on the PVS boundary (artificial case); middle row: no flow in the ECS and $v_{\mathrm{PVS}}$ = 1 μm s^−1^; bottom row: mean flow speed in the ECS $v_{\mathrm{ECS}}=0.038$ μm s^−1^ (permeability $\kappa_{\mathrm{ECS}}$ = $10^{-14}$ m^2^) and $v_{\mathrm{PVS}}$ = 1 μm s^−1^. (C) Volume-averaged solute concentration versus time for the three simulations shown in panel (B). (D) The total solute flux across the boundary between the PVS and ECS at all depths at *t* = 1 h, with positive values indicating flux from the ECS to the PVS, and negative values indicating flux in the opposite direction.

First we consider a simple, circular annulus model consisting of a single arteriole PVS surrounded by a porous region of ECS. A schematic diagram of the computational domain is shown in figure 1A. The width of the PVS is $h_{\mathrm{PVS}}$, and the width of the ECS is $h_{\mathrm{ECS}}$, chosen to be half the average distance to the nearest neighbouring arteriole, thus representing the region of the ECS that is cleared by the central PVS. CSF flows from top to bottom along the PVS, carrying solute out of the computational domain at the bottom surface of the PVS. The boundary conditions at the top, bottom and outer wall of the ECS domain are set to prevent solute transport across the boundaries, except in the case of flow in the ECS (as shown in the bottom row of figure 1B), where solute advection across the outer wall is allowed but diffusion is not. The solute diffusivity is greater in the PVS than in the ECS. Parameter values for the simulations are given in table 1.

**Table 1.** Parameter values for the single-PVS local model.

parameter	lower bound	upper bound
arteriole radius $r_{\mathrm{art}}$ (µm)	20	20
width of the PVS $h_{\mathrm{PVS}}$ (µm)	1	20
width of the ECS $h_{\mathrm{ECS}}$ (µm)	100	500
depth of the ECS L (µm)	1000	1000
mean flow speed in PVS $v_{\mathrm{PVS}}$ (µm s^−1^)	1	20
PVS diffusion coefficient $D_{\mathrm{PVS}}$ (µm^2^ s^−1^)	180	180
ECS diffusion coefficient $D_{\mathrm{ECS}}$ (µm^2^ s^−1^)	62.3	62.3

We illustrate the effect of the clearance of solute by solving the time-dependent Problem 1 assuming a uniform initial concentration C=1 of solute in the ECS. Results of the corresponding simulations are shown in figure 1B,C, where we plot the concentration distribution in the domain at various times and the time dependence of the volume-averaged concentration. In the top two rows of figure 1B, there is no flow in the ECS (Scenario A). The top row shows the distribution when the concentration is artificially maintained at zero along the outer surface of the PVS: this corresponds to instantaneous removal of solute that reaches the PVS boundary, which can be thought of as corresponding to an infinite flow velocity in the PVS. (This zero-concentration boundary condition is sometimes used in brain-clearance models.) In this case, concentration drops rapidly over time, there is no build-up of solute in the PVS, and the concentration distribution is independent of the axial (z) location; diffusion occurs only in the radial direction.

The second row in figure 1B shows the concentration distribution for a finite, realistic flow velocity along the PVS. Solute-free CSF enters the PVS at the top surface of the domain, and the uniform flow velocity is $v_{\mathrm{PVS}}=1$ µm s^−1^. (This value of the velocity is based on simulations in our network model described in §4: see figure 6C.) The solute concentration in the PVS increases monotonically going downstream, as more solute enters from the ECS, so the concentration gradient across the PVS boundary decreases monotonically, and hence the rate of removal of solute from the ECS decreases monotonically downstream. Comparing these plots with those in the top row shows quite clearly the substantial effect of solute build-up in the PVS, which reduces the rate of clearance. Within the ECS, concentration varies in both the axial and radial directions, with concentration increasing with z (into the brain). Thus, there is some back-diffusion in the ECS, in the negative *z*-direction, but this component of the diffusive transport is generally weaker than the radial component, which operates across a shorter distance, until radial clearance is essentially complete. Figure 1D shows that radial diffusion into the PVS is strongest near the inlet and decreases rapidly with increasing depth into the brain.

The third row of figure 1B shows the effect of adding a flow of ISF in the ECS (Scenario B). Here the entering flow speed in the PVS is again 1 µm s^−1^. The flow in the ECS is a purely radial, outward Darcy flow driven by a 1-Pa pressure drop imposed between the outer boundary of the PVS and the outer boundary of the ECS, across which ISF is allowed to flow. The permeability in the ECS is $\kappa_{ECS}=10^{-14}$ m^2^, and the mean flow velocity in the ECS is $v_{\mathrm{ECS}}=0.038$ µm s^−1^. Solute is cleared faster with this slow flow than without it, as is also evident in the decay of concentration over time (figure 1C). Comparing the two curves with $v_{\mathrm{PVS}}=1$ µm s^−1^ in figure 1C, solute is still mainly cleared by flow through the PVS in the case with ISF flow, and the additional clearance is attributed to the ISF flow through the ECS. Figure 1D shows that solute transport into the PVS only occurs near the inlet and advection by ISF dominates radial transport deeper into the brain.^1^

**Figure 2.**
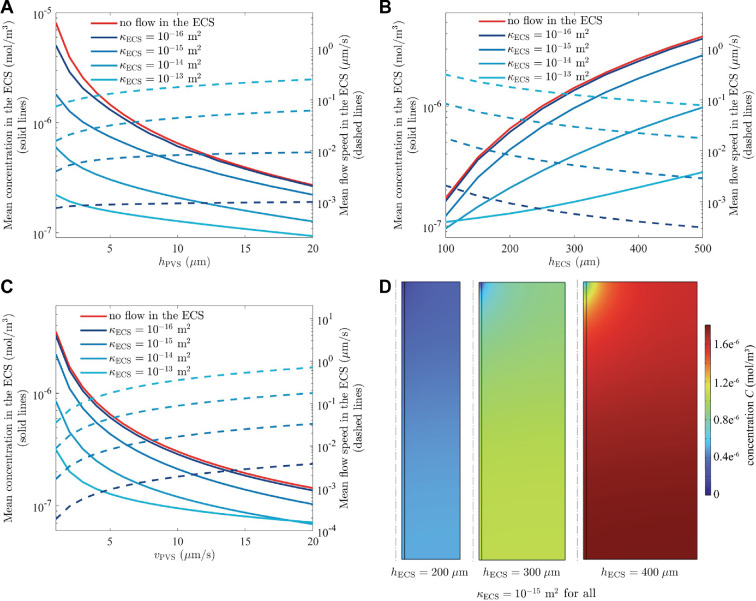
Steady-state (Problem 2) simulations for the single-PVS model (figure 1A). The solute generation rate in the ECS is $f=3.48\times 10^{-11}\;mol\;(m^{-3}\;s^{-1})$ [35]. (A–C) The dependence of the volume-averaged solute concentration in the ECS on the PVS thickness $h_{\mathrm{PVS}}$, ECS thickness $h_{\mathrm{ECS}}$ and PVS velocity $v_{\mathrm{PVS}}$, for the case with no flow in the ECS and several cases with flow in the ECS for different permeabilities ($\kappa_{\mathrm{ECS}}$). In panel (A), $h_{\mathrm{ECS}}$ = 200 μm and $v_{\mathrm{PVS}}$= 5 μm, and increasing $h_{\mathrm{PVS}}$ decreases the mean concentration in the ECS. In panel (B), $h_{\mathrm{PVS}}$ = 10 μm and $v_{\mathrm{PVS}}$ = 5 μm s^−1^, and increasing $h_{\mathrm{ECS}}$ increases the mean concentration in the ECS. In panel (C), $h_{\mathrm{PVS}}$ = 10 μm and $h_{\mathrm{ECS}}$ = 200 μm, and increasing $v_{\mathrm{PVS}}$ decreases the average concentration in the ECS. (D) The concentration distributions for $h_{\mathrm{ECS}}$ = 200, 300, 400 μm, with $\kappa_{\mathrm{ECS}}$ = $1\times 10^{-15}$ m^2^.

For this single-PVS model, we also carried out simulations related to the steady-state Problem 2, assuming a uniform source term f for solute production in the ECS. The properties of amyloid-β were used for the solute, and the generation rate was $f=3.48\times 10^{-11}\;mol\;(m^{-3}\;s^{-1})$ [35]. Figure 2 shows how the average solute concentration depends on the sizes of the PVS and ECS, the CSF flow velocity and the permeability $\kappa_{\mathrm{ECS}}$ of the ECS (which governs ISF flow velocity). The solute concentration distributions plotted in figure 2D show clearly that the concentration is lowest near the PVS, as in figure 1, and much greater for larger values of $h_{\mathrm{ECS}}$. Figure 2B shows the average concentration in the ECS in simulations with varying $h_{\mathrm{ECS}}$, again showing higher concentration when $h_{\mathrm{ECS}}$ is larger. The spacing between arteriole and venule PVSs is a dominant factor in solute clearance: this will be shown more realistically in the array model presented in the next subsection. Here there are two effects at play: the solute production increases with increasing width $h_{\mathrm{ECS}}$ and the flow velocity in the ECS drops off with increasing $h_{\mathrm{ECS}}$ (see figure 2B) because the driving pressure gradient is reduced (i.e. the fixed pressure drop occurs over a greater distance). The mean concentration in the ECS decreases with PVS thickness $h_{\mathrm{PVS}}$ (figure 2A) and with flow velocity $v_{\mathrm{PVS}}$ in the PVS, consistent with the idea that a larger, faster-flowing region of clean CSF clears solute more quickly. This trend demonstrates why the C=0 case in figure 1 produced such rapid clearance: it corresponds to $v_{\mathrm{PVS}}=\infty$.

In our simulations, clearance is promoted by flow not only in PVSs but also in the ECS. As figure 2 shows, the steady-state concentration is highest when flow in the ECS is prohibited and diffusion must act alone. When a flow in the ECS is driven by a pressure drop, the concentration decreases monotonically as the permeability $\kappa_{\mathrm{ECS}}$ is increased, allowing faster flow in the ECS and faster clearance by advection.

### 3.2. An array of arteriole and venule perivascular spaces

**Figure 3.**
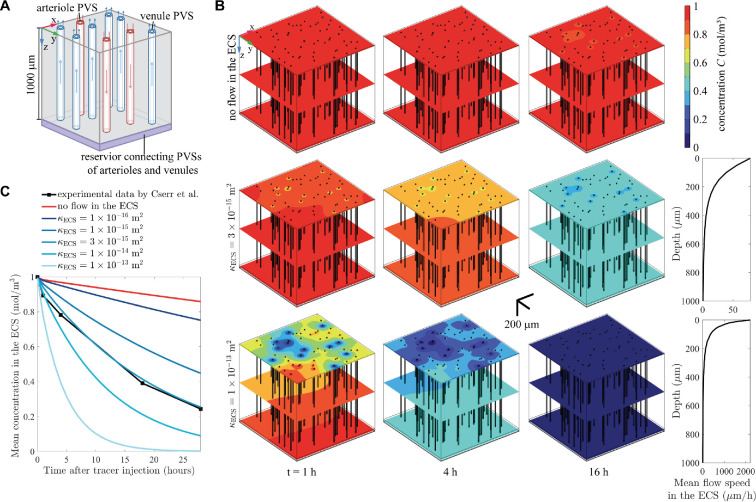
Time-dependent simulations (Problem 1) for an array of PVSs in the mouse brain [36,37]. (A) Schematic of simulation setup: CSF flows through an array of PVSs of arterioles (red) and venules (blue), connected by a reservoir of small volume (purple). The arrows show the flow direction. In most simulations, fluid also flows through the ECS, entering from arteriole PVSs and exiting to venule PVSs. (B) The spatial distributions of the concentration at different times are shown for three simulation cases: no flow in the ECS, $\kappa_{\mathrm{ECS}}$ = $3\times 10^{-15}$ m^2^ and $\kappa_{\mathrm{ECS}}$ = $1\times 10^{-13}$ m^2^. The mean flow speed in the arteriole PVSs is approximately 1 μm s^−1^, while the mean flow speed in the ECS is considerably slower, decreasing rapidly with depth into the brain. (C) The mean concentration and the mean magnitude of concentration gradient in the ECS for time-dependent simulations. The concentration decreases after the tracer is injected. The case with no flow in the ECS greatly overestimates the concentration, and the case with $\kappa_{\mathrm{ECS}}$ = $3\times 10^{-15}$ m^2^ generally fits the experimental data of Cserr *et al*. [19]. For greater values of $\kappa_{\mathrm{ECS}}$ the magnitude of the concentration gradient is initially larger shortly after tracer injection, but it decreases more rapidly over time.

Next we consider a more realistic local model that incorporates an array of arteriole and venule PVSs embedded in a larger region of the parenchymal ECS. Each PVS in the array is a uniform and straight annular cylinder, as in the single-PVS model above, and all the PVSs in the array are parallel. The relative numbers of arteriole and venule PVSs and their spacings in the array are based on experimental data on these arrangements in the mouse brain [36], as modelled by Schreder *et al*. [37]. The dataset used in this study is from the MATLAB file in the supplementary material in [37], Blinder_Coordinates.filtered, with the label ‘au’. As illustrated in figure 3A, fluid flows along the arteriole PVSs, through a small reservoir, and then along the venule PVSs. For this model, in addition to examining Scenario A (no flow in the ECS), we also examine Scenario B by including a flow of ISF in the ECS. The flow in Scenario B is modelled as a Darcy flow in the porous PVSs and the ECS, following a procedure similar to that in [37] but extended to a three-dimensional computational domain. The flow is driven by a pressure difference between the outer surfaces of the arteriole PVSs (which serve as sources of CSF) and the outer surfaces of venule PVSs (which serve as sinks). In the simulations in figure 3B, the pressure at the outer surfaces of arteriole PVSs is 0.1 mmHg and the pressure at the outer surfaces of venule PVSs is zero, and the resulting mean flow speed in the arteriole PVSs is approximately 1 µm s^−1^.

Figure 3B shows the solute concentration at three different times, for simulations with three different values of the ECS permeability $\kappa_{\mathrm{ECS}}$. The concentration decreases with time in all cases, but the decrease is strikingly slow in the absence of flow in the ECS ($\kappa_{\mathrm{ECS}}=0$) and is much quicker with greater permeability. The case with no ECS flow leaves more tracer in the tissue after 16 h than in the moderate-permeability case ($\kappa_{\mathrm{ECS}}=3\times 10^{-15}m^{2}$) after 4 h or the high-permeability case ($\kappa_{\mathrm{ECS}}=1\times 10^{-13}m^{2}$) after just 1 h.

Figure 3C shows the volume-averaged concentration in the ECS as it varies over time, for various values of the ECS permeability $\kappa_{\mathrm{ECS}}$. Consistent with figure 3B, the concentration decreases most slowly in the absence of flow, and the rate of decrease increases with permeability. Also shown in figure 3B is the time decay of the average concentration (normalized by its maximum value) measured in the experiments by Cserr *et al*. [19]. Those researchers infused radio-labelled tracers into rat brains and measured the tracer mass in the CSF after 1, 4, 18 and 28 h. Here we see that the simulation with a permeability of $\kappa_{\mathrm{ECS}}$ = $3\times 10^{-15}$ m^2^ fits the experimental data well. In that simulation, the mean flow speed in the ECS was 11.9 µm h^−1^.

To justify the comparison between our simulation results for the mouse brain and the experimental results for the rat brain by Cserr *et al*. [19], we enlarged the computational domain by 50% in all dimensions to represent the rat brain, in which the pial vasculature covers three times as much area as in mice but has only twice as many penetrating vessels [36]. There is a clear size difference between mouse and rat brains, with the typical volume of a mouse brain being 415 mm^3^ [38] and that of a rat brain being 1765 mm^3^ [39], but the vascular networks in the cortex are similar in mouse and rat brains [36,40] and the venule–arteriole ratio is 2.6 for rodents [41]. Our simulation of the rat brain with $\kappa_{\mathrm{ECS}}$ = $3\times 10^{-15}$ m^2^ showed that the normalized concentration in the ECS after 28 h also matches that measured by Cserr *et al*., and the concentrations in mouse and rat brain simulations differ by less than 5%.

The flow speed in the ECS varies substantially with cortical depth (depth into the brain): near the surface, the mean flow speed is 78.5 µm h^−1^, which is in good agreement with the estimate of 60−190 µm h^−1^ by Bork *et al*. [25]. There, advection is strong and solute is cleared faster than in deeper regions. This creates a concentration gradient (see figure 3B) and enhances solute diffusion towards the surface.

**Figure 4.**
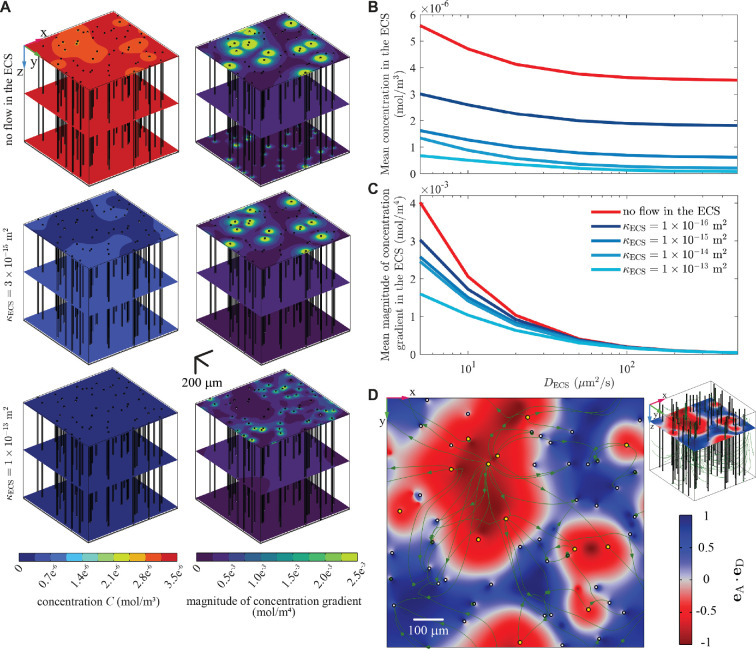
Steady-state simulations of an array of PVSs of arterioles and venules in a mouse brain [36,37]. (A) Concentration (left column) and magnitude of concentration gradient (right column) at three different cortical depths. (B) The mean concentration in the ECS for solutes of different diffusivities. Higher values of $D_{\mathrm{ECS}}$ correspond to solutes with smaller molecular masses. (C) The mean magnitude of the concentration gradient in the ECS for solutes of different diffusivities. (D) Solute flux alignment ${\text{e}}_{\text{A}}\cdot {\text{e}}_{\text{D}}$ at depth *z* = 250 μm for $\kappa_{ECS}$ = $3\times 10^{-15}$ m^2^. The directions of advective and diffusive flux typically differ by >90° near arteriole PVSs and <90° near venule PVSs. Also shown are projections of the streamlines of the ECS flow onto the *z* = 250 μm plane.

Steady-state simulations (Problem 2) with the PVS array were carried out with the same solute generation rate as in the single-PVS simulations, $f=3.48\times 10^{-11}mol\;(m^{-3}\;s^{-1})$. Figure 4A shows the steady-state concentration and the magnitude of the concentration gradient for three cases: when flow in the ECS is prohibited, when the ECS permeability is $\kappa_{\mathrm{ECS}}$ = $3\times 10^{-15}$ m^2^ (the value that gives a good match to the experimental data of Cserr *et al*., as shown in figure 3), and when the ECS permeability is $\kappa_{\mathrm{ECS}}$ = $1\times 10^{-13}$ m^2^. The concentration is highest without ECS flow and lowest when the permeability is greatest, consistent with the idea that advection in the ECS helps clear solute. Steady-state concentration gradients follow the opposite trend, being steepest in the absence of ECS flow and more gradual when ECS permeability is greater and the flow is faster. That is, the model shows that advection facilitates a more uniform biochemical environment for brain tissue.

In addition to the case of amyloid-β, we simulated solutes of different diffusivities to study the effect of molecular mass on solute transport. We assume that changing the molecular mass does not change the advective transport and changes only the solute diffusivities in the PVSs and ECS, and that the diffusivity is inversely proportional to the cube root of the molecular mass, based on the Stokes–Einstein equation. Figure 4B,C shows the variation of the mean concentration and concentration gradient in the ECS with ECS diffusivity, $D_{\mathrm{ECS}}$, for various values of the ECS permeability $\kappa_{\mathrm{ECS}}$. The mean concentration increases as $D_{\mathrm{ECS}}$ decreases, consistent with the fact that lower diffusivity slows clearance. As shown in figure 4A, the steady-state concentration is highest without flow in the ECS and decreases with increasing ECS permeability.

In the ECS, the directions of the advective and diffusive fluxes vary substantially throughout the domain. Figure 4D shows the solute flux alignment, that is, the scalar product of the unit vectors in the directions of the advective and diffusive fluxes (${\text{e}}_{\text{A}}$ and ${\text{e}}_{\text{D}}$, respectively). The two fluxes tend to have directions separated by more than $90^{\circ }$ (${\text{e}}_{\text{A}}\cdot {\text{e}}_{\text{D}}<0$), and thus oppose each other, near arterioles. On the other hand, the two fluxes tend to support each other (${\text{e}}_{\text{A}}\cdot {\text{e}}_{\text{D}}>0$) near venules. Those observations are consistent with diffusion towards all PVSs (since solute concentration is lower in PVSs than in the ECS) occurring simultaneously with advection away from arteriole PVSs and towards venule PVSs (in the direction of ISF flow). (Keep in mind that it is the divergence of these fluxes, not the fluxes themselves, that determines the local rate of solute clearance.)

**Figure 5.**
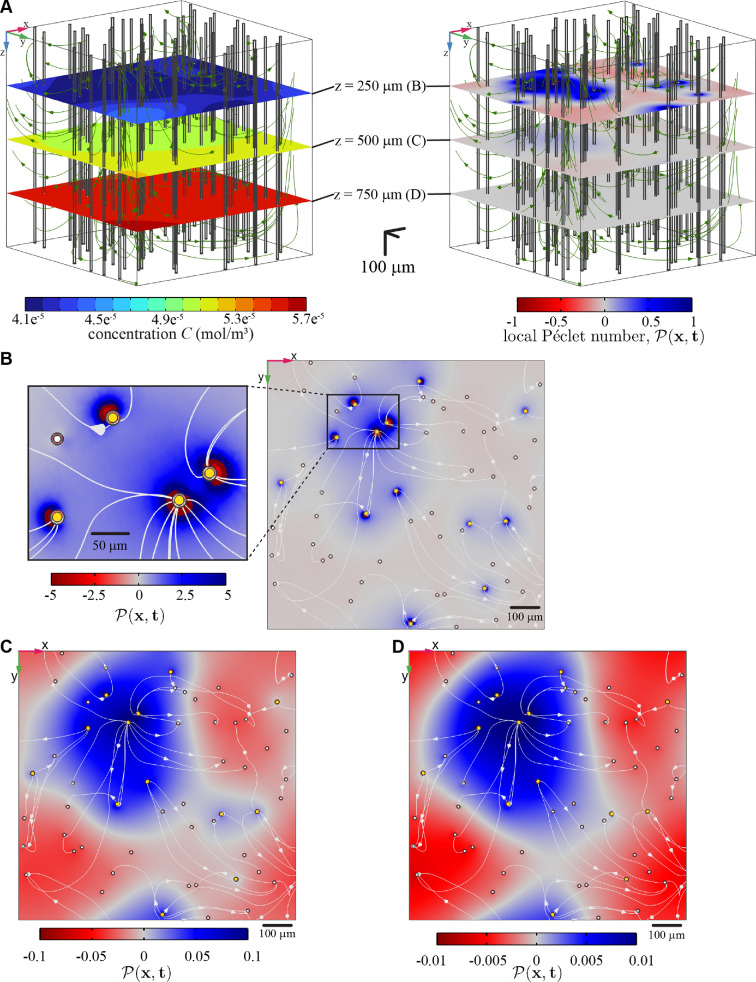
The local Péclet number, P(x), for the steady-state amyloid-β simulation case with $\kappa_{ECS}=3\times 10^{-15}$ m^2^. (A) Concentration distributions and P(x,t) at depths z=250 μm, 500 μm and 750 μm. Streamlines of the ECS flow are also shown. (B–D) P(x) on the same slices, with yellow circles indicating arterioles, white circles indicating venules, and white curves indicating projections of the three-dimensional streamlines plotted in panel (A). Note in panel (B) that |P|>1 near the surface and particularly near PVSs of arterioles (see the enlarged image), indicating that advection is stronger than diffusion there. Far from the surface, |P(x)|≪1, indicating that diffusion is stronger than advection there. Overall, the dominance of advection near the surface sets the clearance pattern, which is quite different from that in the absence of flow in the ECS.

For a steady-state simulation with ECS flow, figure 5 shows the local Péclet number P(x) at three cortical depths. P is of order unity or greater near the surface but much smaller in deeper regions. Note in figure 5B that near the surface and in the immediate surroundings of an arteriole (within about 15 µm), P is typically negative, implying that advection and diffusion act in opposite senses. Near an arteriole, advection is decreasing local concentration by bringing low-concentration fluid from the arteriole PVS, but diffusion is increasing local concentration by bringing solute from other directions, where concentration is higher (compare with figure 4D). However, slightly farther from the arteriole, P typically reverses sign and becomes positive, implying that advection and diffusion act in supporting senses. Beyond that maximum, flow away from the arteriole PVS means that advection tends to increase the local concentration, as does diffusion. By contrast, regions around venules typically show no reversal of P; there, advection and diffusion typically act in supporting senses. Reversals are not evident, either, around arterioles at greater depth (see figure 5C,D). We attribute that observation to two complementary mechanisms: flow there is slower, and solute concentration within the arteriole PVS is not as low.

This simulation shows that, although the local Péclet number P is quite small throughout most of the ECS domain, the fact that its magnitude is larger than unity in small regions near the arterioles is crucial, in that the pattern of solute clearance is completely different from what it would be in the absence of a flow in the ECS. Although in this case a single, global Péclet number for the ECS is small, and the local Péclet number is small throughout most of the ECS domain, it would be misleading to say that diffusion dominates advection in the ECS.

## 4. Global model of fluid transport using a hydraulic network model

**Figure 6.**
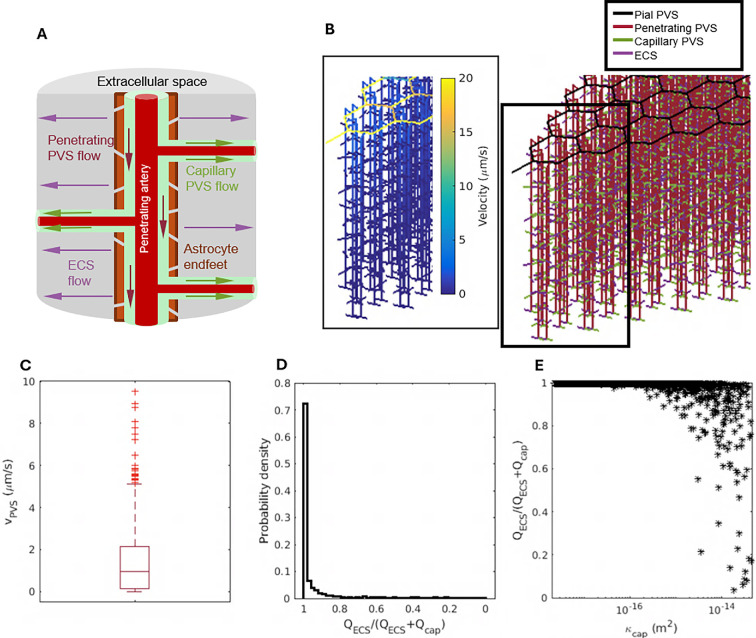
(A) A cylindrical extracellular region around a penetrating arteriole, as modelled. Arrows indicate fluid flow. (B) A sample network geometry, coloured by segment type: perivascular spaces (PVSs) and extracellular space (ECS). The inset shows velocities for the case of ECS permeability $\kappa_{\mathrm{ECS}}=3.0\times 10^{-15}$ m^2^. (C) A boxplot of the mean velocity in the penetrating PVSs from the simulations that allow ECS flow. The boxes identify the interquartile range, with simulations that produced outliers identified by '+'. The median velocity is 1.4 μm s^−1^. (D) Probability density of $Q_{\mathrm{ECS}}/(Q_{\mathrm{ECS}}+Q_{\mathrm{cap}})$, the volume fraction of fluid that flows through the ECS rather than through capillary PVSs. (E) The volume fraction of flow through the ECS depends primarily on $\kappa_{\mathrm{cap}}$, the permeability of capillary perivascular spaces. When $\kappa_{\mathrm{cap}}$ is large, the hydraulic resistance of capillary PVSs is small. In simulations that allow flow through the ECS, fluid moves at approximately 1 μm s^−1^ through penetrating PVSs and flows primarily through extracellular spaces, unless the capillary PVSs have an extremely low hydraulic resistance.

Having found that local solute transport is greatly enhanced by advection in the ECS, we now consider the effect of advection in the ECS on global fluid transport, using a previously published hydraulic network model of glymphatic flow in the mouse brain [42]. This model includes a simplified representation of the pial PVSs, inspired by the vascular model proposed in [36], and a brain-wide configuration of penetrating vessels, as measured in [40] and [43]. The model encompasses nine generations of pial PVSs, with 324 penetrating PVSs branching from the pial surface. Each penetrating PVS is surrounded by a boundary formed by astrocyte endfeet separated by gaps through which fluid can pass. Beyond the gaps lies an axisymmetric, cylindrical region of porous tissue. Penetrating PVSs are also connected to capillary PVSs, through which fluid can alternatively pass, as sketched in figure 6A. (Although the existence of capillary PVSs is uncertain, we include them for completeness.) The inlet of this network represents the PVS of the middle cerebral artery (MCA), and the network simulates flow along PVSs surrounding all vessels that branch off of the MCA, as well as in the adjacent parenchyma [42], spanning approximately one-third of a typical mouse brain. All flows and pressures are modelled as steady.

As a hydraulic resistance model, the pressure drop in a PVS or the ECS is linearly related to the volume flow rate via the resistance to flow through that space. The precise value of hydraulic resistance for a PVSs or ECS segment depends on the parameters described in table 2, and the relationship between parameters and hydraulic resistance values are described thoroughly by [42]. Additionally, the volume flow rate is conserved at bifurcations, connecting the network’s segments and resulting in a solvable system of linear equations. Before these equations can be solved, additional boundary conditions must be applied at the inlet and outlets of the model. The outlets of the model refer to the edges of the capillary PVSs and ECSs, which represent perivenous spaces: the reference pressure is set to zero in all such spaces. Then, we select a pressure drop such that the flow speed along the third of the model closest to the MCA inlet is 18.7 µm s^−1^, to match experimental particle-tracking measurements of flow speed along the MCA in that same region [11]. The result is the calculated flow rate through each PVS and segment of ECS, as well as the corresponding pressure drop for each segment, for a given set of parameters.

**Table 2.** Parameters varied in global simulations, along with their ranges of variation.

parameter	lower bound	upper bound	ref.
fraction pial efflux $E_{\mathrm{pial}}$	0	0.8	[44]
pial PVS area ratio $\Gamma_{\mathrm{pial}}$	0.5	2	[11]
penetrating PVS area ratio $\Gamma_{\mathrm{pen}}$	0.5	2	[11]
penetrating PVS permeability $\kappa_{\mathrm{pen}}$ (m^2^)	$4.50\times 10^{-15}$	$3.71\times 10^{-12}$	[45,46]
capillary PVS permeability $\kappa_{\mathrm{cap}}$ (m^2^)	$2.25\times 10^{-18}$	$4.66\times 10^{-14}$	[47,48]
capillary area ratio $\Gamma_{\mathrm{cap}}$	0.07	2	[49,50]
capillary effective length $L_{\mathrm{cap}}$ (m)	$5.00\times 10^{-5}$	$4.00\times 10^{-4}$	[42]
capillary radius $r_{\mathrm{cap}}$ (m)	$1.50\times 10^{-6}$	$4.5\times 10^{-6}$	[51]
endfoot wall thickness T (m)	$2.00\times 10^{-7}$	$1.00\times 10^{-6}$	[52]
endfoot cavity fraction $F_{c}$	0.003	0.37	[52,53]

While this model lacks the dimensional realism and time dynamics of the local simulations described above, its computational efficiency allows repeated simulations with thousands of different values for parameters, such as the cross-sectional areas and permeabilities of the PVSs and ECSs. We ran 2000 simulations while randomly varying 10 key parameters over the ranges listed in table 2 (which are the same as those used for a prior sensitivity analysis on solute transport following dye injection [54]). By sweeping the ranges of estimated parameter values, we can estimate the average fluid-dynamic behaviour despite the lack of accurate *in vivo* measurements. In the initial simulations, we allowed ECS flow, randomly varying $\kappa_{\mathrm{ECS}}$ between $1.2\times 10^{-17}$ m^2^ [21] and $4.5\times 10^{-15}$ m^2^ [45].

The results of one such simulation are shown in figure 6B, using the value $\kappa_{\mathrm{ECS}}$ = $3.00\times 10^{-15}$ m^2^ that agrees closely with the tracer experiments of Cserr *et al*. [19] (see §3 above). Fluid flows fastest near the inlet, nearly as fast throughout the pial PVSs, and substantially slower in penetrating PVSs and passageways further downstream. In all the simulations allowing ECS flow, the average flow speed in penetrating PVSs is about 1 µm s^−1^, although the flow speed itself can vary by orders of magnitude (figure 6C). Accordingly, we set the flow speed in penetrating arteriole PVSs to be 1 µm s^−1^ in the local-model simulations described in §3.

In our global model, fluid leaving a penetrating PVS must either pass between endfeet and on through the ECS, or else pass along capillary PVSs. In 70% of the global simulations allowing ECS flow, more than 99% of the fluid passes through the ECS instead of the capillary PVSs (figure 6D). This finding suggests that a flow of ISF in the ECS plays a central role in the circulation of water-like fluid in the brain.

**Figure 7.**
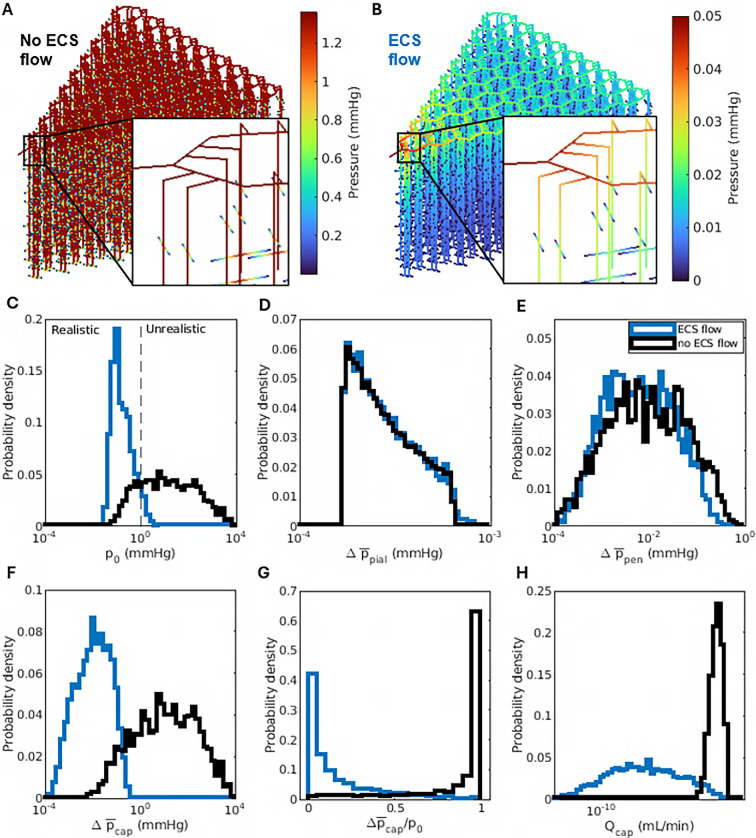
The ECS provides a low-resistance fluid pathway. (A) Pressure in each segment of the PVSs and ECS, in an example simulation where flow in the ECS is prohibited. (B) Pressure in each segment, in a simulation that is identical except that flow in the ECS is permitted ($\kappa_{\mathrm{ECS}}=3\times 10^{-15}$ m^2^). (C) Probability density functions of the global pressure drop $p_{0}$, considering all simulations with and without flow in the ECS. Pressure drops greater than 1 mmHg are considered unrealistic. (D–F) Probability density functions of the mean pressure drop in pial, penetrating and capillary PVSs. (G) Probability density functions of the mean pressure drop in capillary PVSs, as a fraction of the global pressure drop. (H) Probability density functions of total volumetric flow rate through capillary PVSs. Prohibiting ECS flow causes large global pressure drops which occur primarily in capillary PVSs (not in pial or penetrating PVSs) and are a consequence of large flow rates there.

Next, we ran 2000 more simulations, randomly varying the parameters listed in table 2 as before, but prohibiting ECS flow by choosing $\kappa_{\mathrm{ECS}}=0$. Figure 7A shows the pressure in one such simulation.

The total pressure drop $p_{0}$ is large, exceeding 1.3 mmHg. That drop occurs almost exclusively in the capillary PVSs; pressure in pial and penetrating PVSs is nearly uniform. For comparison, figure 7B shows the pressure in a simulation using identical parameters, except that flow is permitted in the ECS ($\kappa_{ECS}=3\times 10^{-15}$ m^2^). There, the total pressure drop is much smaller (0.05 mmHg) and is not concentrated in any part of the network, instead occurring gradually throughout.

The trends apparent in these two examples persisted when we considered all our simulations collectively. First, prohibiting ECS flow correlated with large total pressure drops. Among all simulations prohibiting ECS flow, the average pressure drop across the global model was 249.08 mmHg, much greater than the 0.31 mmHg average for simulations allowing ECS flow. In fact, many simulations without ECS flow had global pressure drops exceeding those in typical simulations allowing ECS flow by orders of magnitude, as shown in figure 7C. Although the pressure drop between pial PVSs and lymphatic vessels has never been measured, its order of magnitude has been estimated to be 1 mmHg [55]. Simulations in which the pressure drop exceeds that value can be considered unrealistic. According to that criterion, 78.3% of the simulations prohibiting ECS flow are unrealistic, compared with just 6.5% of the simulations allowing ECS flow.

Second, the large pressure drops associated with prohibiting ECS flow occurred almost entirely in the capillary PVSs. Figure 7D,E shows that the mean pressure drops across pial and penetrating PVSs did not change appreciably when ECS flow was prohibited. When ECS flow was allowed, the mean pressure drops along pial and penetrating PVS channels were 3.1 × 10^−4^ mmHg and 0.020 mmHg, respectively. When ECS flow was prohibited, the mean pressure drops across pial and penetrating PVS segments became 3.0 × 10^−4^ and 0.037 mmHg. However, figure 7F shows that the mean pressure drop across capillary PVSs was far greater when ECS flow was prohibited than when it was allowed, among all simulations. The mean pressure drop across capillary PVSs was 86.5% of the global pressure drop, on average, when ECS flow was prohibited, but only 16.0% when ECS flow was allowed. Those averages are consistent with the underlying distributions, shown in figure 7G. The existence of large pressure drops in capillary PVSs when ECS flow is prohibited is to be expected because blocking the ECS reroutes fluid through capillary PVSs, resulting in far greater flow rates there (see figure 7H). If capillary PVSs exist, they must be small (see table 2), implying high hydraulic resistance and therefore large pressure drops.

## 5. Discussion

In this study, we have considered advection and diffusion in the cerebrospinal and interstitial fluid filling perivascular and extracellular spaces in the brain, as they relate to the dispersion of an injected tracer and the clearance of metabolic waste. The results of our local models and our global hydraulic network model suggest that a flow of ISF in the ECS is required to match experimental data on tracer movement, interstitial pressure variation and waste clearance.

When there is no flow of ISF (Scenario A), our local models show substantially slower clearance than when ISF flow is included (Scenario B), as shown for example in figure 3C, as well as steeper concentration gradients. In these simulations, the downstream accumulation of solute in the arteriole PVSs substantially slows clearance by impeding diffusion into the PVSs. This accumulation could be reduced by higher flow speeds in the PVSs, but unrealistically high flow speeds are required to produce the rates of clearance observed in experiments.

We find that the usual, overall Péclet number can be a poor indicator of the actual clearance mechanism in the parenchyma because local effects can substantially alter global transport. To address this deficiency, we have introduced a local Péclet number P(x,t), defined as the ratio of the advective and diffusive terms (not characteristic scales) in the advection–diffusion equation. This dimensionless parameter varies spatially and is large in small, localized regions surrounding the PVSs if arteriole and venule PVSs serve as sources and sinks for a slow flow of ISF in the ECS. Those regions can substantially change the clearance pattern. PVSs serve as sources and sinks in the proposed valve mechanism created by the endfoot gaps in the arteriole PVS wall [29–31], in which arteriole pulsations drive a small amount of CSF from the PVS into the ECS and establish a pressure drop between the arteriole and venule PVSs.

Using our multi-PVS local model, we estimate the value of the ECS permeability from the experimental data of Cserr *et al*. [19] by matching the rate at which the average concentration of a tracer decreases with time: we find the approximate value $\kappa_{\mathrm{ECS}}$ = $3\times 10^{-15}$ m^2^ for mouse and rat brains.

According to our global hydraulic network model, the overall pressure drop across the glymphatic system is unrealistically large in most simulations in which ECS flow is prohibited, but realistically small in nearly all simulations in which ECS flow is allowed. Additionally, in most of the simulations in which ECS flow is allowed, nearly all fluid passes through the ECS, not the capillary PVSs. The large overall pressure drops in simulations in which ECS flow is prohibited arise because all fluid is forced through capillary PVSs, small spaces with large hydraulic resistance where flow can proceed only with large driving pressure gradients. From this, we would expect *in vivo* experiments to show substantial pressure gradients in the brain and fast ISF flow around capillaries if there is in fact no ECS flow. Since this fluid behaviour has not been observed experimentally, and very fast PVS flows would likely be observed in MRI, we conclude that slow flows in the ECS must occur instead.

Our results also provide insight into sleep–wake variations in solute transport. The results of our local models show faster solute transport through the ECS when flow rates are higher, but the flow into the ECS results in a concentration profile that depends substantially on depth in the brain. The behaviour of the high resistance, minimal flow case is similar to that in the brain state of an awake animal, suggesting that there is slow, but uniform, transport of nutrients and ions into brain tissue when an animal is awake. When the animal is asleep, the permeability of the ECS increases [28], leading to much faster clearance of naturally produced solutes (figure 3). This variation is in accord with a theoretical scaling analysis of the effects of the changes in ECS permeability that occur from awake to asleep [27].

There are important caveats to this work. Values of several parameters needed to accurately simulate flows in penetrating PVSs are unknown. While the configurations of surface PVSs have been carefully characterized [56,57] and the CSF flow fields within them have been carefully measured [11–14], little is known about penetrating PVSs. In particular, cross-sections and flows in venule PVSs have not been measured. We attempted to overcome this uncertainty by using a range of parameter values to estimate flow speeds in penetrating PVSs with the hydraulic network model. Since solute transport through the ECS is sensitive to changes in the mean velocities in PVSs around arterioles and venules (figure 2C), our simulations may not match *in vivo* clearance if real flows are much faster or slower than 1 µm s^−1^ in penetrating PVSs. Additionally, precise pathways of CSF and ISF flow are not known, and efflux routes are particularly uncertain. We have attempted to compensate by including many plausible pathways in our global models. Finally, much of the brain’s waste is removed not by advection or diffusion but by phagocytosis and/or chemical breakdown. Waste may also be cleared by crossing the blood–brain barrier, though the idea is debatable. We have said little about these alternative clearance mechanisms. That said, all would appear in our models as reaction terms, so if the production rate f is interpreted as the *net* production (taking these other clearance mechanisms into account), the above analysis and discussion holds true.

Solute transport by advection and diffusion in biological systems is complicated by the presence of semi-permeable cell membranes. Solute transport around and through membranes is impeded by the unstirred layer effect [17]. This effect involves an apparent reduction in membrane permeability as solute travelling via advection builds up around a membrane that is more permeable to water than to the solute (as in a sieve). The small, high-concentration region next to the membrane produces an osmotic gradient opposing advection, thus decreasing transport through cells [58,59]. The effect of unstirred layers around cells, which is not accounted for in our models, might reduce solute transport in and out of PVSs, where CSF must flow through small gaps between astrocyte endfeet.

Our findings suggest future work. Our simulations of an array of penetrating perivascular spaces used the locations of vessels in a mouse cortex; performing similar simulations using locations of vessels in a human cortex might reveal the extent to which our findings are conserved across species. There are additional parameters to consider when comparing rodent and human brains and when modelling different physiological and disease states [60]. The fact that rodents have more cortical veins than cortical arterioles, but the reverse is true for primates, suggests that solute transport through the ECS, from arteriole PVSs to venule PVSs, might differ in interesting and important ways. Similarly, our global model represents the domain of the middle cerebral artery in a murine brain. The domain of a human middle cerebral artery is many times larger and involves more generations of bifurcations and daughter vessels, so performing simulations like those described above for a human global model might reveal new behaviours. Finally, experiments to reduce the uncertainty of model parameters would allow more precise predictions than we can yet make. Measuring any of the properties listed in tables 1 and 2 more precisely would help; measuring the permeability of penetrating PVSs is most important [54,61]. Measurements of pressure or concentration gradients could be used immediately to validate and correct models and would have important implications for the field.

## Data Availability

There are no new experimental data involved in our paper. The simulations presented in §3 of the paper were all carried out using the commercial software COMSOL, and the simulations presented in §4 were done using the program for our hydraulic network model, which is available at Zenodo [62].

## Footnotes

^1^
We mention in passing that the results presented in figure 1 are mathematically equivalent to an analogous problem in heat transfer, in which the domain represents a simple heat exchanger, *C* is the temperature, *D* is the thermal diffusivity, and the results show the cooling in the outer annulus.

## Appendix A. Numerical methods for the local models

For the local models, we used the finite element method code COMSOL Multiphysics^®^ 6.1 to perform the numerical simulations. The simulations were performed in two steps: the first step was solving for the fluid velocity field and the second step was calculating the concentration by solving the advection–diffusion equation (2.1). In the local models, the ECS was modelled as a porous medium, but the PVS can be considered as either a porous medium or an open space, and both cases were simulated and compared in this study. When the PVSs were treated as porous media, the flow in the PVS was modelled as a Darcy flow by solving the Darcy equation. When the PVS was treated as an open space, the flow there was modelled as laminar viscous flow by solving the Navier–Stokes equation. For these two different approaches to modelling the fluid flow in the PVS, the variance in average concentration in the ECS was found to be negligible (< 0.22%) when the average flow speeds were kept the same. Therefore, given the lower computational cost, the PVS was considered to be a porous medium in the simulations reported here.

The fluid motion in the porous ECS is governed by Darcy’s law:

(A.1)
$$\text{u}=-\frac{\kappa }{\mu }\nabla p,\;\nabla \cdot \text{u}=0,$$

where u is the velocity field, κ is the permeability, μ is the dynamic viscosity of the fluid and p is the fluid pressure. In all simulations, we used $\mu =0.7\times 10^{-3}$ Pa s, the viscosity of water at 37°C.

We first validated our numerical methods by comparing the numerical results with analytical solutions of one-dimensional advection–diffusion problems, obtaining good agreement. Mesh sensitivity studies were also performed to ensure that the meshes were sufficiently fine to resolve the computational domains and that the numerical results did not change substantially when the mesh size was decreased further. For the two-dimensional axisymmetric cases the mesh size ranged from 1 µm to 5 µm, and for the three-dimensional cases the mesh size ranged from 1 µm to 25 µm.
